# Multilevel composition fractionation process for high-value utilization of wheat straw cellulose

**DOI:** 10.1186/s13068-014-0137-3

**Published:** 2014-11-18

**Authors:** Hong-Zhang Chen, Zhi-Hua Liu

**Affiliations:** State Key Laboratory of Biochemical Engineering, Institute of Process Engineering, Chinese Academy of Sciences, No.1 Zhongguancun North Second Street, Haidian District Beijing, 100190 PR China; University of Chinese Academy of Sciences, No.19A Yuquan Road, Shijingshan District Beijing, 100049 PR China

**Keywords:** Multilevel composition fractionation process, Steam explosion, Ethanol extraction, Wheat straw biomass, High-value utilization, Long fibers, Short fibers, Ionic liquid, Regenerated cellulose film

## Abstract

**Background:**

Biomass refining into multiple products has gained considerable momentum due to its potential benefits for economic and environmental sustainability. However, the recalcitrance of biomass is a major challenge in bio-based product production. Multilevel composition fractionation processes should be beneficial in overcoming biomass recalcitrance and achieving effective conversion of multiple compositions of biomass. The present study concerns the fractionation of wheat straw using steam explosion, coupled with ethanol extraction, and that this facilitates the establishment of sugars and lignin platform and enables the production of regenerated cellulose films.

**Results:**

The results showed that the hemicellulose fractionation yield was 73% under steam explosion at 1.6 MPa for 5.2 minutes, while the lignin fractionation yield was 90% by ethanol extraction at 160°C for 2 hours and with 60% ethanol (v/v). The cellulose yield reached up to 93% after steam explosion coupled with ethanol extraction. Therefore, cellulose sugar, hemicellulose sugar, and lignin platform were established effectively in the present study. Long fibers (retained by a 40-mesh screening) accounted for 90% of the total cellulose fibers, and the glucan conversion of short fibers was 90% at 9.0 hours with a cellulase loading of 25 filter paper units/g cellulose in enzymatic hydrolysis. Regenerated cellulose film was prepared from long fibers using [bmim]Cl, and the tensile strength and breaking elongation was 120 MPa and 4.8%, respectively. The cross-section of regenerated cellulose film prepared by [bmim]Cl displayed homogeneous structure, which indicated a dense architecture and a better mechanical performance.

**Conclusions:**

Multilevel composition fractionation process using steam explosion followed by ethanol extraction was shown to be an effective process by which wheat straw could be fractionated into different polymeric fractions with high yields. High-value utilization of wheat straw cellulose was achieved by preparing regenerated cellulose film using [bmim]Cl.

## Introduction

Biomass refining has gained considerable momentum due to its potential benefits for economic and environmental sustainability and given the current challenges of energy security [1,2]. Lignocellulosic biomass (LCB) is a plentiful and renewable resource for biofuels, biomaterials, and biochemicals. However, LCB has evolved complex structural and chemical mechanisms for hindering microorganisms from attacking its structural sugars [1,3]. The cellulose of LCB has a crystalline structure that is water insoluble and resistant to depolymerization. It is connected to hemicellulose and lignin via a hydrogen bond, while hemicellulose connects to lignin via a covalent bond, which forms the lignin-carbohydrate-complex (LCC). These are known as biomass recalcitrance, which affects liquid penetration in pretreatment and enzyme accessibility and activity in hydrolysis, and hence conversion costs [3]. Because of the recalcitrance of biomass and the lack of systematic theories on biomass refining, there is still a certain bottleneck before the large-scale utilization of LCB is viable. In LCB conversion, a multilevel fractional conversion process should be feasible for the commercial utilization of LCB due to its intrinsic characteristic of multiple components. In this process, many technologies should be integrated for the directional multilevel fractionation and conversion of LCB based on its intrinsic properties [1,4].

Owing to the recalcitrance of biomass, pretreatment is a crucial step which alters the structure of biomass in order to make carbohydrate polymers more accessible to the enzymes. Steam explosion (SE) has generally been recognized as one of the most effective pretreatments for breaking down the LCC structure [1,5,6]. It offers several advantages including use of less hazardous chemicals and greater potential for energy efficiency compared with other pretreatments [6,7]. Among various organic solvents, ethanol extraction is a clean delignification method due to the low toxicity [1,5,8]. Furthermore, ethanol extraction has many other advantages including the easy recovery of solvent and separation of valuable products due to the high volatility [9].

Regenerated cellulose films (RCF) are widely applied in ultrafiltration, dialysis, and the release of pharmacon. Chemical processing of cellulose is extremely difficult in general because cellulose cannot be liquidized and is insoluble in common solvents due to its highly developed hydrogen bond networks and partially crystalline structure [10-12]. Cellulose dissolution in traditional industrial process requires relatively harsh conditions as well as a variety of expensive chemicals [10]. Currently, cellulose films are prepared by cellulose acetate hydrolysis or chemical derivatization, dissolution, and regeneration, which are technically complex and pollute the environment [11,12]. Ionic liquids (ILs) are designated as desirable ‘green’ solvents due to their low volatility, excellent dissolution ability, and high thermochemical stability, which have currently attracted widespread attention [10,13,14]. RCF preparation from LCB using ILs should be the subject of accelerating interest and awareness.

In the present work, a multilevel composition fractionation process for wheat straw, based on steam explosion followed by ethanol extraction, was investigated. A specific objective was to study the production of regenerated cellulose films from cellulose recovered in the process. Steam explosion (SE) and ethanol extraction conditions that might affect composition fractionation performance were carried out. Enzyme kinetics of short fibers was compared with that of raw material and steam-exploded wheat straw (SEWS). Two kinds of RCF were prepared from wheat straw cellulose (WSC) long fibers using [bmim]Cl and aqueous NaOH. The mechanical performance of RCF, including tensile strength and breaking elongation, was determined and the micro-structure of RCF was also examined by scanning electron microscopy (SEM).

## Results and discussion

### Composition of raw material and characteristic of cellulose fibers

The biomass conversion efficiency for bio-based products mainly depends upon the physic-chemical property of raw material (RM) [1,15]. Table 1 shows that the compositions of RM varied widely among different species. The cellulose content of wheat straw (WS) was higher than that of rice straw (RS) and poplar. In general, the high cellulose content was beneficial to bioethanol production and cellulose film preparation [16,17]. The hemicellulose content of WS was approximate to that of RS. However, the hemicellulose content of WS and RS were about 1.45 times higher than that of poplar. Hemicellulose is the second most common polysaccharide in LCB, but it can be easily degraded in SE and is difficult to be converted by microbes. Thus, developing an effective bioprocess for large-scale conversion of hemicellulose to biofuels and other value-added products is necessary [18]. It should be noticed that the lignin content of WS was 1.16 times higher than that of RS, however it was only three quarters that of poplar. Lignification was an important factor for the recalcitrance of plant cell wall against polysaccharide utilization [19,20], which indicated that the fractionation of lignin should be essential for overcoming biomass recalcitrance. The ash content of WS, which was approximate to that of poplar, was only a quarter of RS. The pretreatment efficiency and the product quality, such as RCF, are closely correlated to the salts generated from the plant cell and attachments [21-23].

**Table 1:** **Chemical components of wheat straw compared with rice straw and poplar**

**Raw material**	**Cellulose**	**Hemicellulose**	**Lignin**	**Ash**
Wheat straw	44.2 (1.8)	26.5 (1.9)	22.4 (1.7)	2.8 (0.6)
Rice straw	36.1 (2.6)	27.2 (3.2)	19.7 (2.5)	12.1 (2.8)
Poplar	39.2 (1.1)	18.8 (1.4)	29.6 (1.6)	1.5 (1.2)

The composition content of raw materials was based on the dry matter. Standard deviations are shown in parentheses.

**Figure 1:**
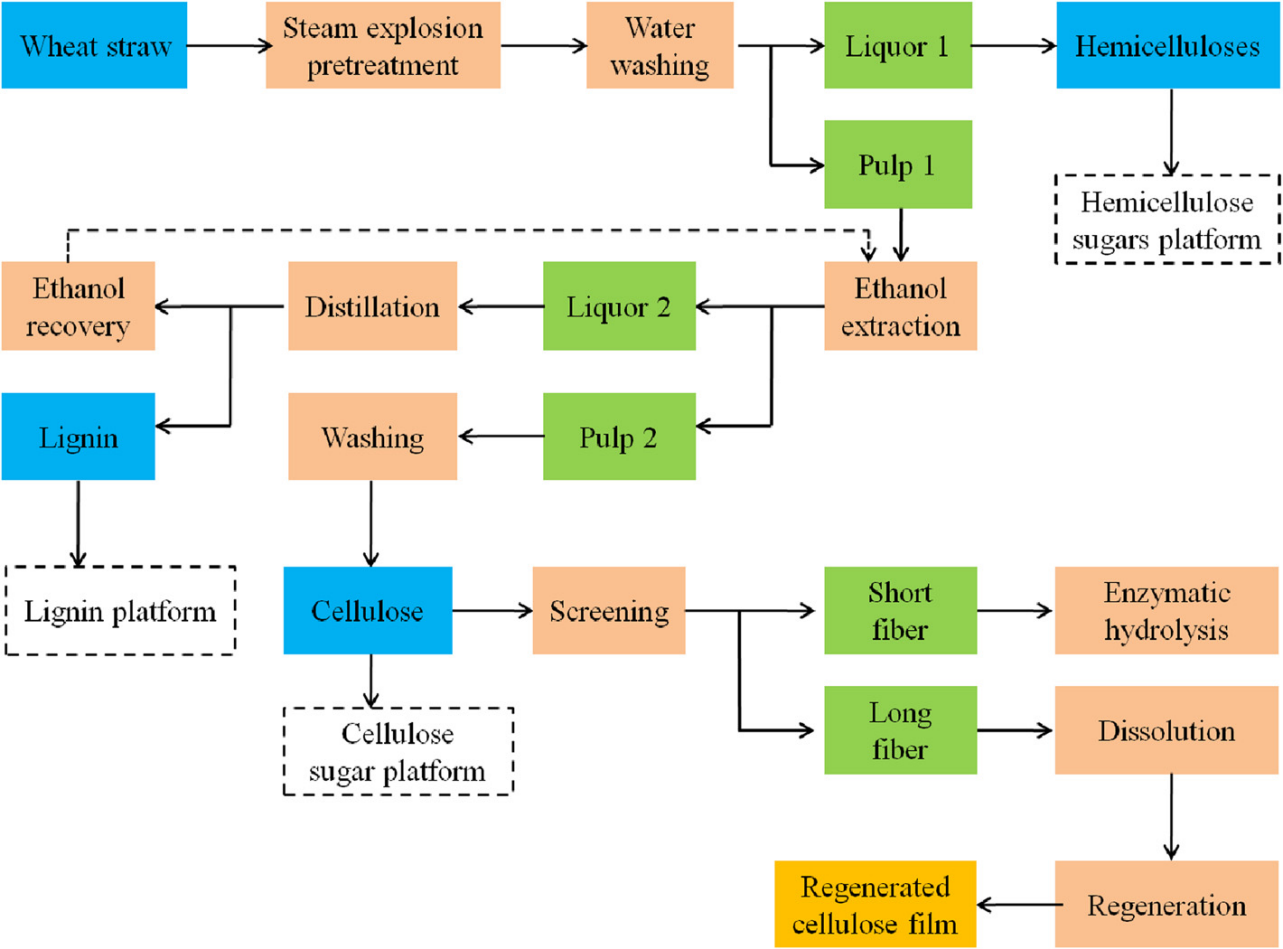
**Flow diagram of the multilevel composition fractionation process (MCFP) of wheat straw (WS) and regenerated cellulose film (RCF) preparation from the long fibers of wheat straw cellulose (WSC).**

The length and width of cellulose fibers are the most important factors affecting RCF preparation [24]. The fiber length and width of WS, RS, and poplar are given in Table 2. It is worth noting that the maximum fiber length of WS was 1.4 times higher than that of RS, but was similar to that of poplar. The average fiber length of WS was 1.4 times and 1.2 times higher than that of RS and poplar, respectively. The general distribution of WS fiber length was also higher. Although the maximum and average fiber width of WS was lower than that of poplar, they were higher than that of RS. The general distribution of fiber width for WS was approximate to that for poplar, while it was higher than that for RS. WS and RS had a higher length-to-width ratio compared with poplar, which reflected the different cellulose fibers characteristics of varied RM. High length-to-width ratio of cellulose should be beneficial to the mechanical property of RCF [23,24]. Therefore, the analysis of composition and fiber length and width of RM suggests that WS should be suitable for biomass refining and RCF preparation. In the present study, a flow chart of the multilevel composition fractionation process (MCFP) of WS and RCF preparation from WSC is given in Figure 1.

**Table 2:** **Fiber length and width of wheat straw compared with rice straw and poplar**

**Raw material**	**Fiber length (mm)**				**Fiber width (μm)**				**Length:width**
	**Maximum**	**Minimum**	**General distribution**	**Average**	**Maximum**	**Minimum**	**General distribution**	**Average**	
Wheat straw	2.94	0.61	1.03-1.60	1.32	24.5	7.4	9.3-15.7	12.9	102
Rice straw	2.13	0.26	0.47-1.43	0.92	17.2	4.3	6.0-9.5	8.1	114
Poplar	2.88	0.48	0.54-1.57	1.11	36.7	6.6	9.1-14.2	14.8	75

### Hemicellulose fractionation by steam explosion

**Figure 2:**
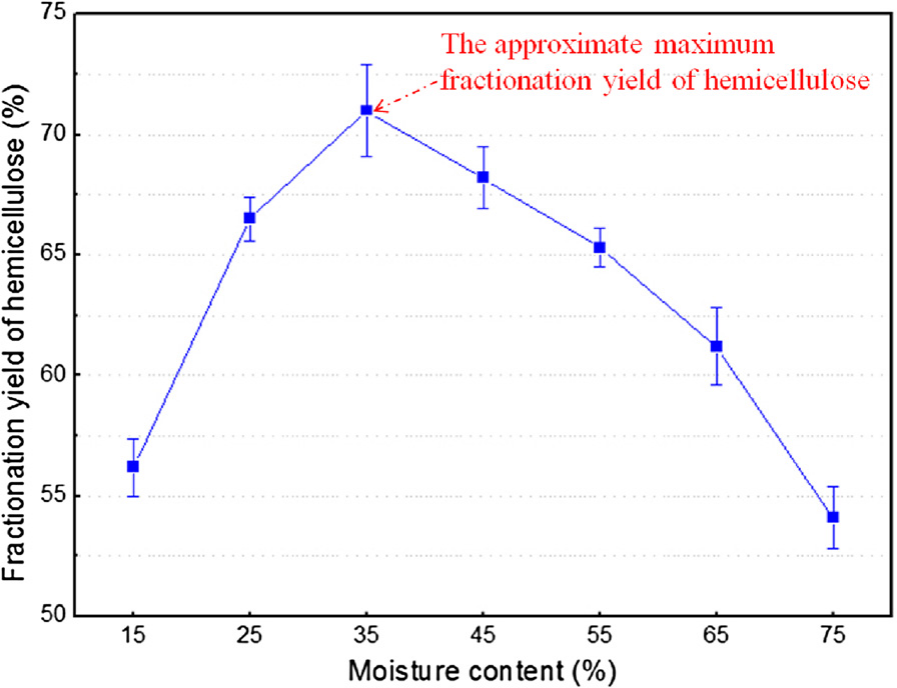
**Effect of moisture content (MC) on hemicellulose fractionation yield during steam explosion (SE) of wheat straw (WS).** Line bars in figure represent standard deviation.

SE without the addition of chemicals was used to fractionate hemicellulose and break the LCC structure of WS. Initial moisture content (MC) is the principal factor governing steam consumption and affecting the fractionation efficiency [25,26]. The effects of MC on the hemicellulose fractionation yield are given in Figure 2. The results showed that the hemicellulose fractionation yield was less than 65% when initial MC was less than 15% or more than 55%, which implied that the excessively low or high MC adversely affected the hemicellulose fractionation performance. However, the maximum fractionation yield of hemicellulose (71%) was obtained under 35% MC, which was chosen as the optimal initial MC.

The effects of SE factors, including pressure (x_1_) and time (x_2_), on hemicellulose fractionation yield were investigated by quadratic rotation-regressive experimental design under 35% MC (Table 3). The regression equation of the hemicellulose fractionation yield (y), pressure (x_1_), and time (x_2_) was obtained by the Matlab R2012 (The MathWorks, Inc. Massachusetts, United States) as follows:

**Table 3:** **Quadratic rotation-regressive design of steam explosion pretreatment and hemicellulose fractionation yield under different pretreatment conditions**

**Test number**	**Pressure x**_**1**_ **(MPa)**	**Time x**_**2**_ **(minutes)**	**Fractionation yield of hemicellulose (%)**
1	1 (2.0)	1 (6)	60.3 (1.7)
2	-1 (1.0)	1	58.3 (1.4)
3	1	-1 (2)	63.2 (1.1)
4	-1	-1	56.3 (2.3)
5	0 (1.5)	1.414 (6.828)	72.7 (1.2)
6	0	-1.414 (1.172)	52.4 (1.6)
7	1.414 (2.207)	0 (4)	63.5 (2.1)
8	-1.414 (0.793)	0	55.1 (1.8)
9	0	0	67.1 (1.6)
10	0	0	67.5 (1.3)
11	0	0	67.1 (1.2)
12	0	0	67.4 (1.4)
13	0	0	67.2 (1.5)

Standard deviations are shown in parentheses.

1 $$ y=67.2482+2.5827{x}_1+3.4882{x}_2-4.3337{x_1}^2-1.245{x}_1{x}_2-2.7082{x_2}^2 $$

The corresponding analysis of variance (ANOVA) value is presented in Table 4. It was clearly observed that pressure and time acted as significant factors affecting hemicellulose fractionation (*P* <0.05). The results showed that the optimal conditions were x_1_ = 0.2125 and x_2_ = 0.5952, of which corresponding pressure and time was 1.6 MPa and 5.2 minutes, respectively. Under the optimal conditions, the maximum fractionation yield of hemicellulose was 73%. Our previous studies also confirmed that SE obviously loosened and disrupted the rigid and highly ordered structure of biomass and partially melted the lignin, which was beneficial to the subsequent conversion process [6,21].

**Table 4:** **ANOVA value obtained from quadratic rotation-regressive design employed in optimization of hemicellulose fractionation yield during steam explosion pretreatment**

**Factor**	**Sum of squares**	**Degree of freedom**	**Mean square**	**Freedom ratio**	***P*** **value**
Corrected model	427.018	7	61.003	47.982	0.000
x_1_	144.993	3	48.331	38.015	0.003
x_2_	237.313	3	79.104	62.220	0.005
Error	6.357	5	1.271		
Corrected total	433.375	12			

After SE pretreatment, SEWS solid was washed and separated from the liquor. The hemicellulose sugars in the liquor were recovered by water countercurrent extraction and decolored with chelating ion exchange resin D412. Our previous research showed that the hemicellulose recovery was about 80% and xylose content accounted for 86.3% [5]. Degradation products in the pretreated liquor, such as acetic acid, 5-hydroxymethylfurfural (HMF), and furfural, were obviously lower in SE compared with other pretreatments [6,8,27]. Thus, hemicellulose was efficiently fractionated from WS by SE and the hemicellulose sugar platform was established (Figure 1).

### Lignin fractionation by ethanol extraction

**Figure 3:**
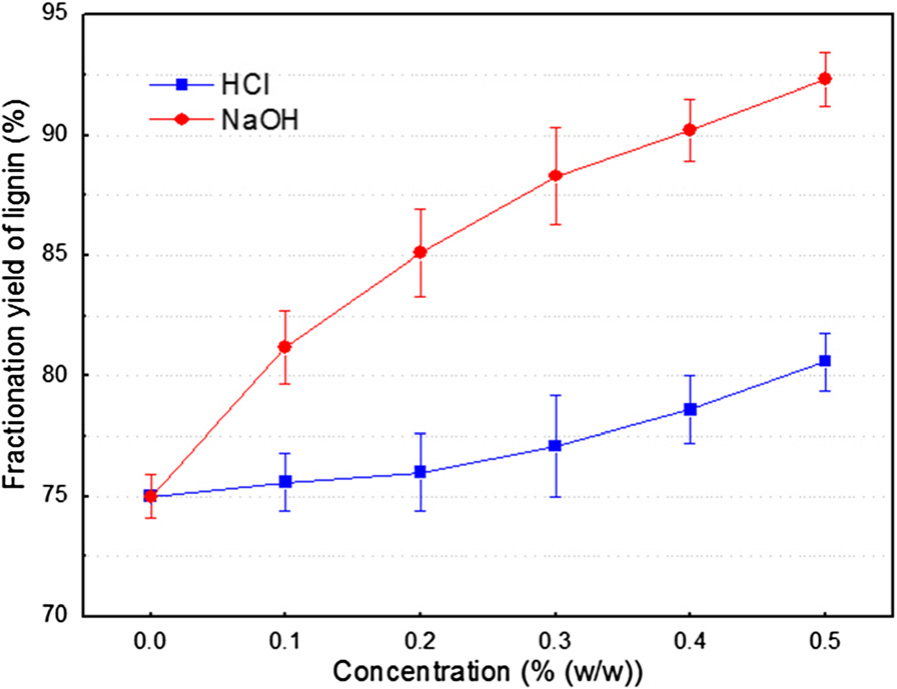
**Effects of HCl and NaOH addition on lignin fractionation yield during ethanol extraction of steam-exploded wheat straw (SEWS).** Line bars in figure represent standard deviation.

Ethanol extraction is a clean delignification method compared with other organic solvents [28,29]. During the ethanol extraction process, 0.1 to 0.5% (w/w) HCl or NaOH was added in order to improve the lignin fractionation efficiency from SEWS (Figure 3). The results showed that the lignin fractionation yield increased by more than 18% with NaOH addition, and less than 4% with HCl addition, with the concentration increasing from 0.1 to 0.5% (w/w), respectively, compared with no NaOH or HCl addition. It should be noted that the lignin fractionation yield with NaOH addition was 5 to 10% more than that with HCl addition, which implied that NaOH obviously improved the ethanol extraction performance. Furthermore, NaOH had no harmful effect on the reactor compared with HCl. Therefore, the optimal condition of NaOH addition was 0.5% (w/w).

The influence of ethanol extraction factors, including ethanol concentration (x_1_), temperature (x_2_), and time (x_3_), on lignin fractionation yield was carried out by means of a 4^3^ orthogonal design with 0.5% (w/w) NaOH addition (Table 5). The corresponding ANOVA value in Table 6 indicated that the order effects of factors on the lignin fractionation yield were temperature (x_2_) > time (x_3_) > ethanol concentration (x_1_). It was clearly observed that temperature (x_2_) and time (x_3_) acted as significant factors affecting lignin fractionation yield (*P* <0.05), and the effect of temperature was more significant than that of other factors. The optimal conditions of ethanol extraction were 160°C for 2 hours with 60% ethanol (v/v), and the corresponding lignin fractionation yield was 90%. Our previous study showed that the delignification rate was 82.3% under an ethanol concentration of 40%, a fiber:liquor ratio of 1:50 (w/v), and a severity log (R) equal to 3.657 (180°C for 20 minutes) [5].

**Table 5:** **Design of orthogonal experiment (L16 (4** ^**3**^ **)) for ethanol extraction of steam-exploded wheat straw and lignin fractionation yield under different conditions**

**Test number**	**Ethanol concentration X**_**1**_ **(%)**	**Temperature X**_**2**_ **(°C)**	**Time X**_**3**_ **(minutes)**	**Fractionation yield of lignin (%)**
1	45	120	60	75.4 (1.3)
2	45	140	120	78.7 (2.1)
3	45	160	150	83.5 (1.6)
4	45	180	90	75.8 (2.1)
5	60	120	90	77.4 (2.3)
6	60	140	150	76.7 (1.8)
7	60	160	120	88.4 (1.6)
8	60	180	60	80.3 (1.2)
9	75	120	120	82.9 (2.4)
10	75	140	60	74.9 (1.6)
11	75	160	90	81.4 (1.9)
12	75	180	150	77.5 (1.5)
13	90	120	150	78.4 (2.1)
14	90	140	90	82.5 (2.0)
15	90	160	60	83.8 (2.3)
16	90	180	120	85.3 (1.1)

Standard deviations are shown in parentheses.

**Table 6:** **ANOVA value obtained from orthogonal design employed in optimization of lignin fractionation yield during ethanol extraction of steam-exploded wheat straw**

**Factor**	**Sum of squares**	**Degree of freedom**	**Mean square**	**Freedom ratio**	***P*** **value**
Corrected model	206.775	9	22.975	4.854	0.034
X_1_	39.842	3	13.281	2.806	0.131
X_2_	94.012	3	31.337	6.621	0.025
X_3_	72.921	3	24.307	5.135	0.043
Error	28.399	6	4.733		
Corrected total	235.174	15			

Ethanol extraction removed the lignin and hemicellulose remaining in the SEWS solid. After the delignification of SEWS, the liquid fraction was separated from the solid fraction. Lignin in the liquid fraction was separated and purified by gradient acid precipitation. Lignin recovery reached 75% under pH 4.0 at 10 minutes sedimentation and the fine lignin recovery was about 85% of the crude lignin after purification. Our previous study also confirmed that the high value-added application of lignin was developed by gradient acid precipitation based on the lignin molecular weight from alkali-extraction liquid fraction of SE stalk [5,30]. The results implied that ethanol extraction resulted in an effective fractionation of lignin, and the lignin platform was established by ethanol extraction (Figure 1). The solid fraction was washed with deionized water until a colorless solution appeared and WSC was obtained. The cellulose yield after SE and ethanol extraction was 93.2% under optimal conditions, which meant that the cellulose sugar platform was also established after MCFP (Figure 1).

### Screening of wheat straw cellulose and enzymatic hydrolysis of short fibers

**Figure 4:**
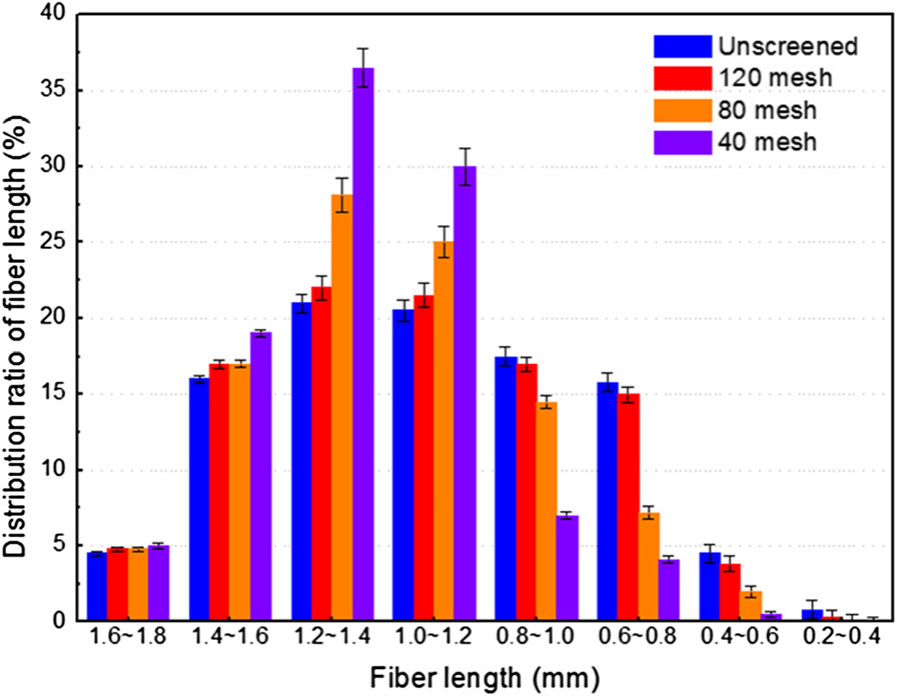
**Effect of screen mesh on the fiber length distribution ratio of wheat straw cellulose (WSC) after steam explosion (SE) and ethanol extraction.** Line bars in figure represent standard deviation.

The biomass cellulose fibers were inhomogeneous and their length distribution varied with RM types (Table 2). In order to prepare RCF, WSC was screened into long fibers and short fibers. The fiber length distribution ratio of WSC under different screen meshes is given in Figure 4. The results showed that the fiber length distribution ratio hardly changed under 120 mesh compared with unscreened WSC, and the screened short fibers were less than 20% of the total short fibers. However, the screening efficiency of long fibers increased with the decrease of screen mesh. It should be noted that fiber length was mainly 1.0 to 1.8 mm under 40 mesh, which accounted for approximately 90% of the total cellulose fibers. The composition analysis showed that cellulose content in screened WSC under 40 mesh was 95.3%, while the ash content was less than 0.6%. These results indicated that the length and purity of WSC screened under 40 mesh should meet the requirements of RCF preparation.

**Figure 5:**
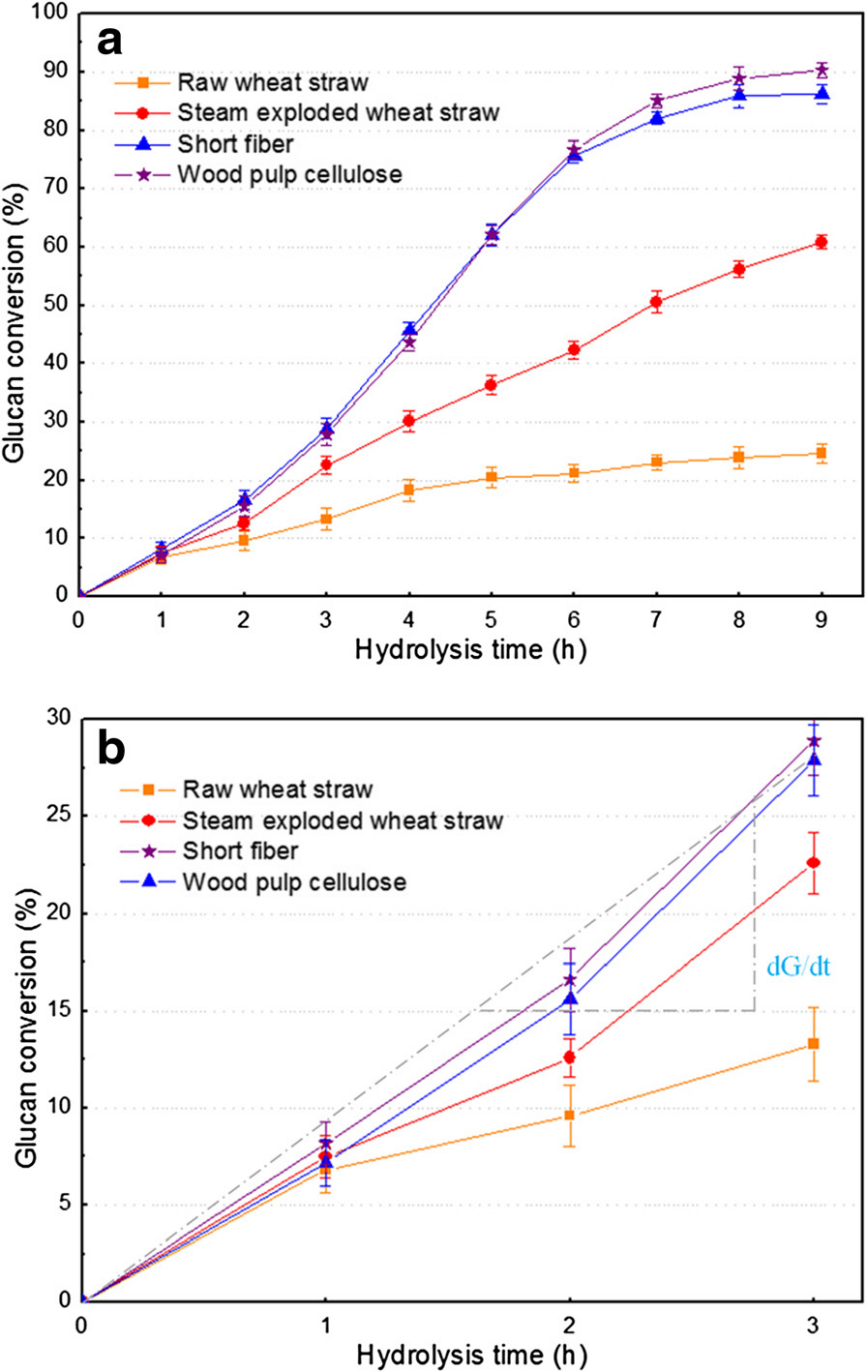
**Enzyme kinetics of the short fibers of wheat straw cellulose (WSC) compared with other substrates (raw wheat straw, steam-exploded wheat straw, and wood pulp cellulose).** Figure 5**a** shows the glucan conversion between 0 and 9 hours hydrolysis, while Figure 5**b** shows the glucan conversion between 0 and 3 hours hydrolysis. The initial hydrolysis rate (dG/dt) was calculated based on the hydrolysis that occurred in the first 3 hours. Line bars in figure represent standard deviation.

About 90% of short fibers were separated from WSC by 40-mesh screening. The cellulose content of short fibers was 93.6%, while the ash content was about 1.1%. Short fibers were not suitable for RCF preparation due to the high content of the epidermis, parenchyma, and other miscellaneous cells [11,31]. Furthermore, the high ash content was another obstacle in RCF preparation. However, short fibers should be effectively converted into glucose by enzymatic hydrolysis. The results in Figure 5a show that glucan conversion slowly increased with hydrolysis time for raw wheat straw (RWS), however it was three times higher for SEWS (60%) than RWS at 9.0 hours, which implied that SE obviously improved the enzymatic hydrolysis performance. The glucan conversion of short fibers was approximate to that of wood pulp cellulose (WPC), and it rapidly increased with the hydrolysis time from 1.0 to 6.0 hours, reaching up to 90% at 9.0 hours, with a cellulase loading of 25 filter paper units (FPU)/g cellulose. The glucan conversion of short fibers was 4.5 times higher than that of RWS at 9.0 hours. It was also interesting to note that the initial hydrolysis rate of short fibers was about 2.5 times and 1.4 times higher than that of RWS and SEWS, respectively (Figure 5b). Therefore, SE coupled with ethanol extraction obviously improved the enzymatic hydrolysis performance of WS. It may be due to the fact that the LCC structure was disrupted and the non-adsorption of the enzyme on lignin was removed, and hence a more accessible area of cellulose for the enzyme was provided in enzymatic hydrolysis.

### Characterization of regenerated cellulose film

**Figure 6:**
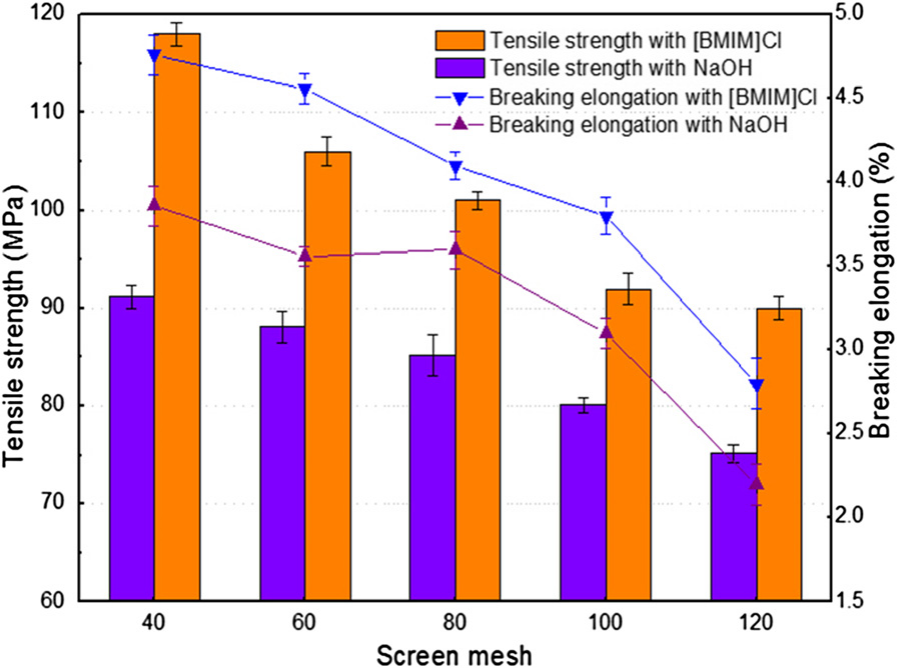
**The tensile strength and the breaking elongation of regenerated cellulose films (RCF) prepared by [bmim]Cl and NaOH under different screen meshes.** Line bars in figure represent standard deviation.

In order to evaluate the suitability of WSC for biomaterial preparation, RCF were prepared by dissolving and regenerating in [bmim]Cl and NaOH under different screen meshes. Tensile strength and breaking elongation, which reflect the deformation of a material under an external force, are used as the key metrics for RCF mechanical performance assessment [12,32]. Figure 6 shows that the tensile strength of RCF prepared by [bmim]Cl was 1.20 to 1.32 times that prepared by NaOH, with screen mesh size decreasing from 120 to 40, while the breaking elongation was 1.27 to 1.31 times that prepared by NaOH. The results indicated that the RCF prepared by [bmim]Cl exhibited better mechanical performance than that prepared by NaOH. Furthermore, [bmim]Cl has several advantages including a low melting point, low vapor pressure, and wide liquid range, and it can be recycled after regeneration. Therefore, [bmim]Cl should be a suitable solvent for RCF preparation [33]. Figure 6 also showed that tensile strength and breaking elongation increased by 33% and 75% for [bmim]Cl, and by 23% and 68% for NaOH, with screen mesh size decreasing from 120 to 40, respectively. The results suggested that smaller screen mesh resulted in the better mechanical performance of RCF due to the fact that the short fibers were removed and the purity of long fibers was improved.

**Figure 7:**
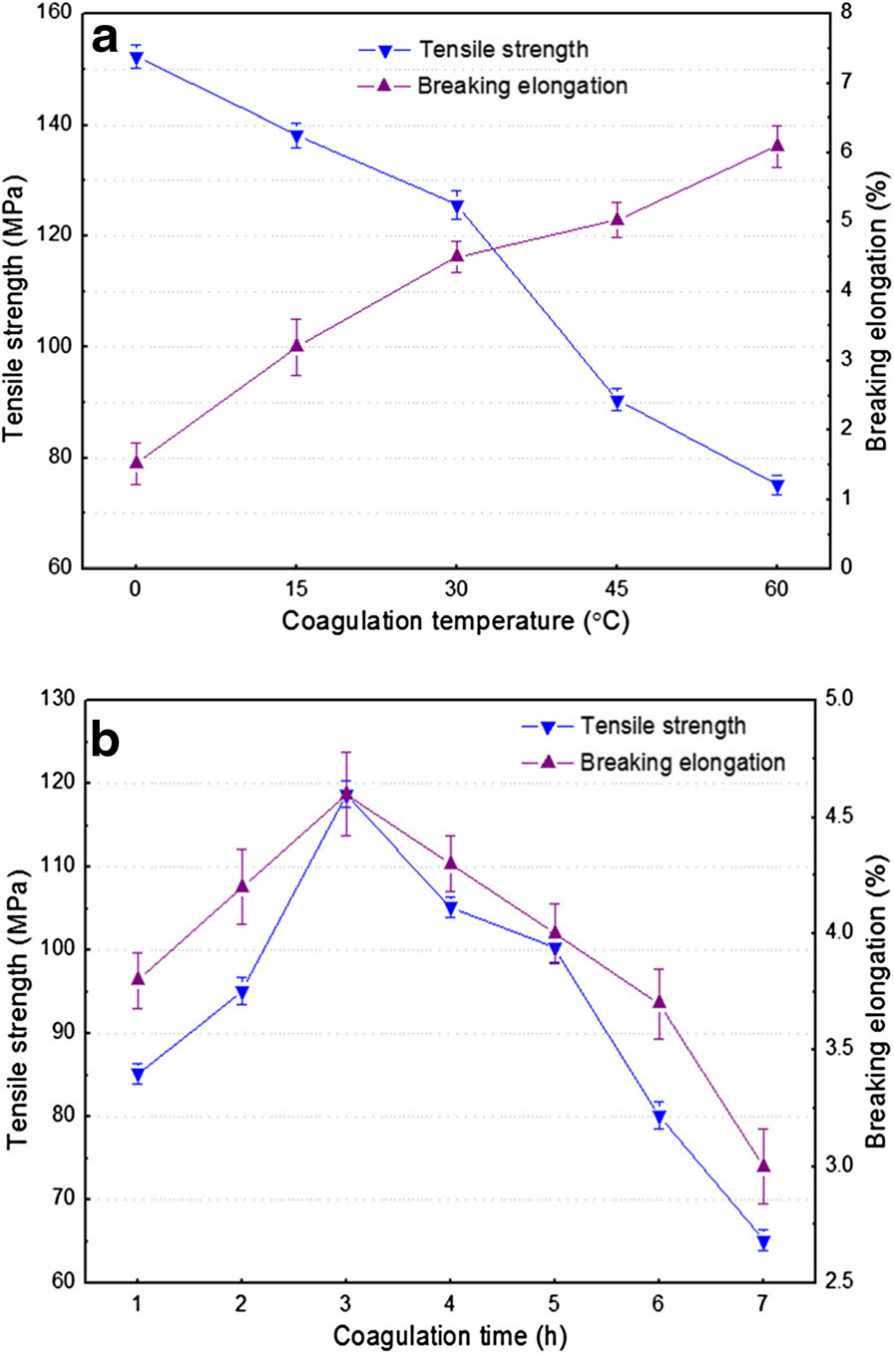
**Effects of coagulation temperature (a) and coagulation time (b) on the tensile strength and the breaking elongation of regenerated cellulose films (RCF) prepared by [bmim]Cl.** Line bars in figure represent standard deviation.

The effects of coagulation temperature and coagulation time on the tensile strength and breaking elongation of RCF prepared by [bmim]Cl were investigated (Figure 7). Figure 7a presented that the tensile strength of RCF obviously decreased with the increase of coagulation temperature, however, breaking elongation showed the opposite trend. The main reason for this may be that the degree of polymerization (DP) of cellulose long fibers decreased with the increase of coagulation temperature because strong polar chloride ions attacked the intermolecular and intramolecular hydrogen bonds of cellulose molecules at high temperature, which may break cellulose chains to varying degrees and lead to the degradation of cellulose DP. The low DP resulted in low tensile strength and high breaking elongation due to the crosslinked action of the cellulose long fibers during RCF preparation [12]. Figure 7b presented that both tensile strength and breaking elongation were obviously increased, with coagulation time varying from 1.0 to 3.0 hours, and then apparently decreased with coagulation time varying from 3.0 to 7.0 hours. The possible reason for this phenomenon was that the crosslinked action of cellulose long fibers was enhanced at initial coagulation time, but the cellulose DP may decrease with the increase of coagulation time, which exhibited an adverse effect for RCF mechanical performance [12]. Therefore, considering both tensile strength and breaking elongation, the optimal coagulation temperature and time for RCF preparation should be 30°C and 3.0 hours, respectively.

**Figure 8:**
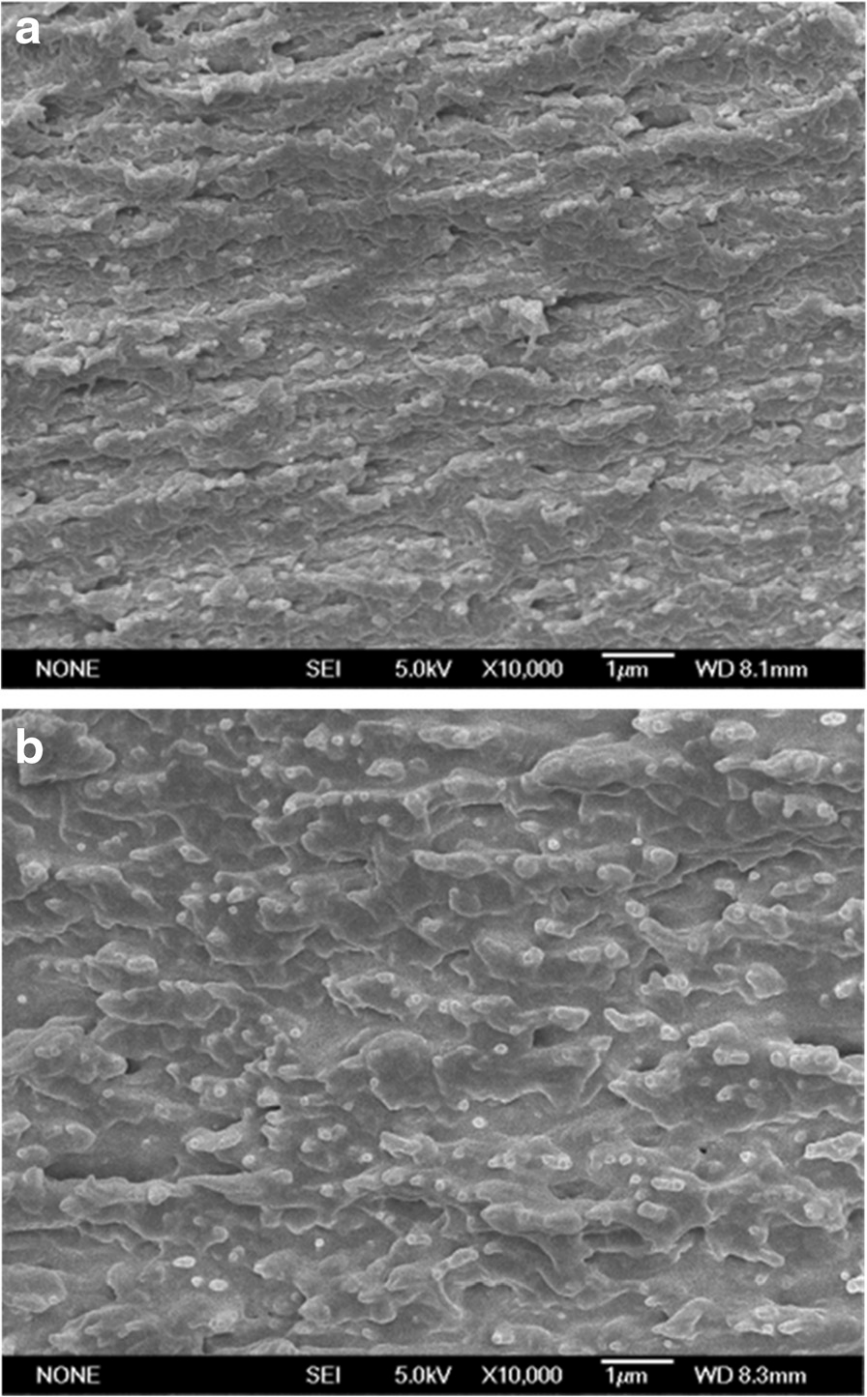
**SEM images of the cross-sections of regenerated cellulose films (RCF) prepared by [bmim]Cl and NaOH. (a)** The cellulose film was regenerated from [bmim]Cl; **(b)** the cellulose film was regenerated from NaOH.

The microstructure of the cross-section of RCF prepared by [bmim]Cl and NaOH was examined by SEM, and the images are presented in Figure 8. Figure 8a showed that the RCF prepared by NaOH displayed a microporous structure in the inner section, and that the pore size was larger than that of RCF prepared by [bmim]Cl. The structure of RCF prepared by NaOH seemed loose, which may explain the low mechanical performance. Although the cross-section exhibited a microporous structure, the RCF prepared by [bmim]Cl displayed a homogeneous structure, indicating a dense architecture and a good mechanical performance (Figure 8b). The above results were consistent with the tensile strength and breaking elongation analyses. Therefore, the RCF prepared from WSC using [bmim]Cl should be a promising biomaterial in a separate field, which indicates that the high-value utilization of WSC can be achieved by preparing RCF.

The properties of several RCFs prepared by different methods are given in Table 7. The results showed that the tensile strength and breaking elongation of RCF prepared by [bmim]Cl were obvious higher than that by NaOH, which was similar to the above discussion. The tensile strength and breaking elongation of RCF prepared from WSC were slightly lower than that from WPC. A possible reason for this result was that WSC had a lower DP compared with WPC. The mechanical properties decreased with the decrease of DP due to the fact that the low DP resulted in the low degree of fibers orientation and cellulose crystallinity, and hence the low crosslinked action efficiency. However, WS is a plentiful and accessible agricultural waste, and converting WS into RCF using [bmim]Cl could be beneficial to both the agriculture economy and the environmental crisis. Therefore, it is expected that WS should be a potential source for RCF preparation.

**Table 7:** **Characteristics of several regenerated cellulose films prepared by different methods**

**Preparation method**		**Tensile strength/MPa**	**Break elongation ratio/%**
**Solvent**	**Cellulose source**		
[bmim]Cl	wood pulp cellulose	163.9 - 181.7	5.7 - 6.9
	wheat straw cellulose	101.2 - 120.1	4.1 - 4.8
NaOH	wood pulp cellulose	128.7 - 136.4	4.7 - 5.4
	wheat straw cellulose	75.2 - 90.0	3.6 - 3.8

Tensile strength ranges shown are approximate.

### Multilevel composition fractionation process analysis

Biomass has evolved complex structural and chemical mechanism for resisting assaults on its structural sugars from the microbe, which is key to reducing the LCB conversion cost [3,34,35]. In order to obtain a clean and economical conversion process of LCB, a number of breakthroughs are needed, not only in individual process steps, but also in the balance and combination of these processes [1]. The conversion process of multiple products should consider the intrinsic characteristic of LCB itself [34,35]. Therefore, in order to overcome biomass recalcitrance and prepare RCF, MCFP was systematically investigated based on the multiple compositions of WS in the present study.

SE without the addition of chemicals was used as the core technology for the fractionation process of WS [1]. Hemicellulose as polysaccharides accounts for a large proportion of biomass. The most important biological role of hemicellulose is its contribution to strengthening the cell wall by interaction with cellulose and lignin, which implies that the fractionation of hemicellulose from LCB should be essential for breaking biomass recalcitrance and improving the LCB conversion efficiency [3]. SE was the most effective pretreatment on hemicellulose fractionation which was attributed to its chemical effects and mechanical force effects. There was an auto-hydrolysis stage and an explosion stage after the penetration of high-pressure steam into the plant cell wall [6,21]. At the auto-hydrolysis stage, hemicellulose was thought to be hydrolyzed into monomeric, oligomeric sugars and partial furfural by auto-hydrolysis effects with the acetic and other acids catalysis derived from acetyl groups at high temperature. The ester linkages between carbohydrates and lignin was disrupted, and hence lignin was melted, solubilized, and recondensed [21,36]. At the sudden explosive decompression stage, the biomass particles were exploded into small pieces, and the LCC structure was further disrupted. The fractionation of hemicellulose and the disruption of tight biomass structure were helpful in exposing the cellulose surface and overcoming the biomass recalcitrance. More than 96.3% of cellulose and almost all lignin were retained in the SEWS solid. However, the severity of SE should be controlled in order to avoid the excessive degradation of hemicellulose and cellulose. After SE, hemicellulose could be efficiently recovered through countercurrent extraction and the water was reused for generating high-pressure steam. Therefore, the fractionation of hemicellulose from WS by SE and the building of hemicellulose sugar platform should improve the hemicellulose conversion performance for bio-based products such as xylitol, furfural, and ethanol (Figure 1).

Lignification is a major factor affecting the biomass conversion process. Ethanol extraction has attracted much attention and showed to have a potential use in the delignification of LCB [28]. Delignification by ethanol extraction was a composite process, including degradation, melt, and solubilization of lignin. After SE, lignin was more soluble in ethanol because phenolic hydroxyl buried in lignin was exposed and the important ester linkages between carbohydrates and lignin were seriously disrupted [5,6,21]. Furthermore, the higher solubility of lignin fragments in ethanol-based liquors helped to increase the lignin fractionation yield and reduce lignin condensation [28]. Lignin platform was established by acid precipitation and purification, which can be converted into different potential bio-based products, such as phenolic resin and polyurethane. After ethanol extraction, ethanol was distilled and recovered at a low temperature.

Cellulose as a linear polysaccharide is the most abundant renewable organic material with a host of current and potential uses [12,37]. After SE coupled with ethanol extraction, cellulose was separated and purified and cellulose sugar platform was established, which facilitated the conversion of cellulose into biofuels and RCF. In previous studies, RCF was predominantly produced by the well-known viscose process and the N-methylmorpholine-N-oxide (NMMO) process [12,38,39]. Compared with traditional process, ILs were nonflammable and recyclable and can effectively dissolve cellulose without any activation or pretreatment steps at room temperature [10,11]. RCF was prepared by [bmim]Cl from WSC in the present study. The highest tensile strength of RCF reached up to 152.3 MPa, which was superior to that prepared from cornhusk cellulose by [Amim]Cl (120 MPa) and cotton linters by NaOH/Urea (106 MPa) [12,32]. The mechanical properties of RCF should be above the minimum threshold for certain applications [32], which indicated that RCF prepared by [bmim]Cl had the potential to be used for dialysis, ultrafiltration, and other separation fields. The results suggested that RCF preparation by ILs using relatively cheap WS as a cellulose resource was a feasible method, and hence high-value utilization of WSC can be developed.

In the present study, the three main compositions of WS biomass (cellulose, hemicellulose, and lignin) were effectively fractionated based on SE followed by ethanol extraction. The cellulose sugar platform, hemicellulose sugar platform, and lignin platform had been established, respectively. RCF was produced using [bmim]Cl from WSC recovered in the MCFP, which showed a good mechanical performance. Therefore, the MCFP of WS using SE coupled with ethanol extraction technology facilitates the establishment of sugars and lignin platform and enables the production of RCF.

## Conclusions

Our research suggests that SE coupled with ethanol extraction is an effective process that allows for the fractionation of WS into polymeric fractions with high yields. The maximum hemicellulose fractionation yield was 73% in SE, while the maximum lignin fractionation yield was 90% in ethanol extraction. The cellulose yield after SE and ethanol extraction was 93.2% under the optimal conditions. The glucan conversion of short fibers was 90% at 9.0 hours with a cellulase loading of 25 FPU/g cellulose. RCF was prepared from long fibers by [bmim]Cl and exhibited an excellent mechanical performance. Therefore, MCFP of WS using SE followed by ethanol extraction was an effective method for the fractionation of multiple components and the preparation of RCF.

## Materials and methods

### Fractionation process diagram

A flow chart of multilevel composition fractionation process (MCFP) is given in Figure 1. The chipped WS biomass was pretreated by SE and subsequently washed using deionized water at 75°C to fractionate hemicellulose. Delignification of SEWS was carried out by ethanol extraction. WSC was separated from lignin by filter and then WSC was sieved into long fibers and short fibers. Long fibers were used for RCF preparation, while short fibers were hydrolyzed.

### Raw material preparation

RM including WS, RS, and poplar biomass used in the present study was collected from Chinese Academy of Agriculture Sciences and Tongzhou district in Beijing, China. RM was air-dried to the moisture content of between 5 and 10%. For composition analysis, RM was milled by knife mill (MQF-420, BJZKRF, Beijing, China), and then passed through a screen of 2.0 mm (Shangyu road instrument factory, Zhejiang, China). Composition analysis was carried out according to the Laboratory Analysis Protocol (LAP) of the National Renewable Energy Laboratory (NREL, United States) [40-42].

### Steam explosion

WS was manually cut into 5.0 cm pieces and adjusted to 15 to 75% moisture content (MC) using deionized water before SE. The SE experiment design is shown in Table 3. A total of 400 g chopped WS (DM) was fed into a 4.5-L SE reactor (Weihai Automatic Control Co. Ltd., Weihai, China) by inlet valve. The inlet valve was then closed and the steam valve was turned on. When the steam pressure of the reactor and residence time reached to the target setting, the outlet valve was instantaneously opened and WS was exploded into the reception chamber. After pretreatment, SEWS was washed using deionized water with a liquid-to-solid ratio of 10:1 at 75°C. Solid fraction was then separated from liquid fraction by vacuum filtration using a Buchner funnel. Compositions of the liquid fractions were determined by HPLC.

### Ethanol extraction

The washed SEWS was delignified by ethanol extraction in a mechanical stirring reactor (GSF, DLZK, Beijing, China). The ethanol extraction experiment design is shown in Table 5. A total of 200 g SEWS (DM) was put into the reactor with different ethanol volume fractions, and 0.1 to 0.5% (w/w) HCl or NaOH was added to adjust pH. After ethanol extraction, the remaining cellulose was removed by vacuum filtration. Ethanol was recovered from filtrate by distillation and lignin was recovered at different acidic conditions.

### Enzymatic hydrolysis of short fibers

The long fibers of WSC were separated from short fibers using different size sieves (Shangyu road instrument factory, Zhejiang, China). The demarcation point of long fibers and short fibers was 1.0 mm length in the present study.

Cellulase preparation (Cellic CTEC 2) was purchased from Novozymes (china) Biotechnology Co., Ltd. (Tianjin, China). The cellulase activity of Cellic CTEC 2 is 108 FPU/mL. The short fibers were hydrolyzed at 2% (w/w) solid loading in a 0.05 M citrate buffer solution (pH 4.8) (Sinopharm Chemical Reagent Co.,Ltd, Shanghai, China) with a cellulase loading of 25 FPU/g cellulose. The hydrolysis conditions were 50°C and 200 rpm, and mixtures were sampled at different hydrolysis times. Hydrolysate was separated from the solid residue by centrifugation at 12,000 rpm for 8 minutes, and analyzed by HPLC (Agilent 1200, Agilent Technologies, Santa Clara, California, United States). All these experiments were conducted in triplicate.

### Synthesis of ionic liquid [bmim]Cl

A total of 240 mL 1-butane chloride and 128 mL 1-methylimidazole were added to a 1.0 L round-bottomed flask fitted with a reflux condenser. The mixture was heated to 90°C in an oil bath and maintained for 24 hours with vigorous stirring. It was continuously aerated with nitrogen to prevent oxidation of 1-methylimidazole. After reaction, the excess 1-butane chloride was removed by vacuum distillation. The solvent was cooled and crystallized, and then washed and recrystallized. Water in synthesized [bmim]Cl was removed by vacuum drying. The melting point of [bmim]Cl is around 67°C, and the reaction equation of synthesized [bmim]Cl is:

(2)

### Preparation of regenerated cellulose film

The long fibers sieved from WSC were dried at 65°C for 3 hours in a vacuum oven. A total of 6.0 g long fibers were added to 100 mL [bmim]Cl in a 500 mL flask, and then maintained under 90°C in an oil bath using N_2_ as the shield gas. The mixture was stirred until the long fibers completely dissolved into [bmim]Cl, and the viscous and amber-colored WSC/[bmim]Cl solution was then obtained. RCF was prepared according to the phase inversion process using water as the precipitant. The hot WSC/[bmim]Cl solution was transferred onto a glass plate and covered with another glass plate to control film thickness within a certain range by adjusting the pressure. It was then immediately immersed in distilled water precipitant at different temperatures. The covered glass plate was slowly taken out after film concretion under different concretion times, and washed repeatedly with deionized water to remove residual [bmim]Cl. The film was dried in 40°C vacuum oven for 24 hours. The colorless and transparent RCF was obtained and the [bmim]Cl was recycled and reused in the present study.

### Characterization of regenerated cellulose film

The mechanical properties of dried RCF, including tensile strength (σ) and breaking elongation (ε), were measured by a minicomputer tensile strength tester (XD-125D, Shanghai Xinrenda Company, Shanghai, China). The length and width of samples were 50 mm and 10 mm, respectively. The experiments were carried out at a constant crosshead speed of 40 mm/min. The microstructure of the fracture surface of RCF was examined by SEM (S-3200, HITACHI, Tokyo, Japan). RCF samples were first frozen in liquid nitrogen and then fractured to expose the cross-sectional morphologies.

### Analytical methods

The compositions were analyzed by HPLC (Agilent 1200, Agilent Technologies, Santa Clara, California, United States) equipped with a refractive index detector and an Aminex HPX-87H carbohydrate analysis column (Bio-Rad, Hercules, California, United States) at 65°C with 5 mM H_2_SO_4_ as the mobile phase at a flow rate of 0.6 mL/min. Hemicellulose sugars includes xylose, arabinose, galactose, and mannose in the present study.

Hemicellulose fractionation yield in SE, lignin fractionation yield in ethanol extraction, glucan conversion of short fibers in enzymatic hydrolysis, and the breaking elongation of RCF were calculated as follows:

3 $$ Hemicellulose\  fractionation\  yield\ \left(\%\right)=1- Hemicellulose\  in\  steam\  exploded\  wheat\  straw/ Hemicellulose\  in\  raw\  material\times 100\% $$

4 $$ Lignin\  fractionation\  yield\ \left(\%\right)=1- Lignin\  in\  extracted\  steam\  exploded\  wheat\  straw/ Lignin\  in\  raw\  material\times 100\% $$

5 $$ Glucan\  conversion\ \left(\%\right)= Glucose\  in\  hydrolysate \times \left(162/180\right)/ Glucan\  in\  in itial\  solid\times 100\% $$

6 $$ Breaking\  elongation\ \left(\%\right) = The\  tensile\  length\  of\  regenerated\  cellulose\  film/ The\  initial\  length\  of\  regenerated\  cellulose\  film\times 100\% $$

## Abbreviations

DM: Dry matter
DP: Degree of polymerization
FPU: Filter paper unit
ILs: Ionic liquids
LCB: Lignocellulosic biomass
LCC: Lignin-carbohydrate-complex
MC: Moisture content
MCFP: Multilevel composition fractionation process
RCF: Regenerated cellulose film
RM: Raw material
RS: Rice straw
RWS: Raw wheat straw
SE: Steam explosion
SEM: Scanning electron microscopy
SEWS: Steam exploded wheat straw
WPC: Wood pulp cellulose
WS: Wheat straw
WSC: Wheat straw cellulose
